# Spectrophotometeric Determination of Cefuroxime Axetil from bulk and in its tablet dosage form

**DOI:** 10.4103/0250-474X.41468

**Published:** 2008

**Authors:** M. V. Shinde, S. A. Pishawikar, H. N. More

**Affiliations:** Department of Pharmaceutical Chemistry, Bharati Vidyapeeth College of Pharmacy and Research Center, Near Chitranagari, Kolhapur-416 013, India

**Keywords:** Cefuroxime axetil, yellow orange complex, spectrophotometry, 1-nitroso-2-napthol

## Abstract

A simple rapid spectrophotometric method has been developed for estimation of cefuroxime axetil from bulk drug and tablet dosage form by using 1-nitroso-2-napthol and sodium hydroxide. The method is based on the formation of yellow-orange coloured complex of drug with 1-nitroso-2-napthol having absorbance maxima at 424 nm. The Beer’s law is obeyed in the concentration range of 10-50 μg/ml of the drug but more precisely it obeys in the range of 10- 30 μg/ml. The slope and intercept values are 0.0101 and 0.0838, respectively. Results of analysis of this method were validated statistically and by recovery studies. The method is applied to the marketed tablet formulation. Result of analysis of tablet formulation given as percentage of label claim ±standard deviation is 99.17±1.57. The precision and accuracy was examined by performing recovery studies and was found to be 99.50±1.82. Sandell’s correlation coefficient is calculated as 0.4434. The developed method is simple, sensitive and reproducible and can be used for routine analysis of cefuroxime axetil from bulk and tablet dosage form.

Cefuroxime is chemically (6R,7R)-3-carbamoyloxymethyl-7-[(Z)-2-(2-furyl)-2-(methoxyimino)acetamido]-ceph-3-em-4-carboxylic acid. Cefuroxime is official in Indian pharmacopoeia. It is the first of the series of alpha methoxyiminoacyl substituted cephalosporins that constitute most of the third generation agents available for clinical use. It is active against some beta lactamase strains that are resistant to cefamandole. The literature survey revealed that various methods of analysis for cefuroxime alone or in combination with other drugs have been reported, which included, HPLC^1^–^4^, electrokinetic^5^, HPTLC^6^ and spectrophotometric methods^7^.

The method developed is based on the formation of diazo complex of cefuroxime axetil with 1-nitroso-2-napthol in the presence of sodium hydroxide to give yellow orange coloured chromogen with λ_max_ 424 nm. Reaction conditions were optimized to obtain maximum colour intensity. The method is simple, reproducible and requires low cost and method is applied successfully to the analysis of the marketed tablet formulation.

A Double beam JascoV-530 Model spectrophotometer having 2 matched cells with 1-cm light path was employed for spectral measurements. The tablet dosage form was procured from local market. Sodium hydroxide (0.006 N) was prepared by alligation method. 1-nitroso-2-napthol (25 mg) was weighed accurately and dissolved in 25 ml with double distilled water. Cefuroxime axetil (5 mg) was weighed accurately and dissolved in double distilled water to produce a 100 μg/ml solution.

Working standard solution containing 100 μg/ml of cefuroxime axetil was prepared in double distilled water. Cefuroxime axetil was treated with 1-nitroso-2-napthol and sodium hydroxide leading to formation of yellow- orange coloured complex. The analyte gave maximum absorbance at 424 nm. In this method volume of 1-nitroso-2-napthol (concentration 0.001 mg/ml) was optimized to 2.5 ml.

Using various normality ranges from 1 N to 0.006 N the normality of sodium hydroxide was optimized. Sodium hydroxide of normality equivalent to 0.006 N was found to yield reproducible results. Beer’s law is obeyed in concentration range of 10 to 30 μg/ml. The slope and intercept values are 0.0101 and 0.0838. The correlation coefficient was found to be 0.9990. To study the recovery of cefuroxime axetil, drug from the tablet sample was taken to which different quantities of pure drug (reference standard) was added within the analytical concentration range in the proposed method. The added quantity of individual drug was estimated in the method. % concentration ±SD and coefficient of variance for cefuroxime axetil bulk drug (AS) and cefuroxime axetil recovery sample were found to be 100.27± 1.63,99.50±1.82 and 1.138, 1.1134, respectively. From these values it seems that method is accurate and reproducible for both bulk drug and formulation.

The film coated marketed tablet formulation with 500 mg of drug claim are used for applying developed method on formulation. Twenty tablets of marketed drug were weighed and powdered. The powder equivalent to 5 mg of cefuroxime axetil was weighed accurately and treated with double distilled water (50 ml) to produce 100 μg/ml of the drug solution. The mixture was sonicated for 15 min and filtered through Whatmann filter paper No. 40. The dilutions were made accordingly to concentration range in the given procedure. % concentration ±SD and coefficient of variance for the tablet formulation was found to be 99.17±1.57 and 1.139, respectively.

In this method cefuroxime axetil reacts with 1-nitroso-2-napthol and sodium hydroxide to give yellow orange coloured complex with stability up to 2 h. The complex exhibit maximum absorbance at 424 nm. The Beer’s law was obeyed in the concentration range of 10 to 30 μg.

These reaction conditions were optimized to obtain maximum colour intensity. Proposed method was use for pure bulk drug as well as for marketed formulation. The result obtained compared favorably with labeled amount of drug as well as that of the formulation. None of the usual diluent, lubricant, film formers employed in preparation of tablet dosage form was found to interfere in the proposed procedure. The proposed method hence is specific, precise, accurate and reliable.
